# Dietary supplementation with Essential-oils-cobalt for improving growth performance, meat quality and skin cell capacity of goats

**DOI:** 10.1038/s41598-018-29897-3

**Published:** 2018-08-02

**Authors:** Zhaomin Lei, Ke Zhang, Chao Li, Jianping Wu, Delmer Davis, David Casper, Hui Jiang, Ting Jiao, Xiaolong Wang, Jianfu Wang

**Affiliations:** 1 College of Animal Science and Technology, Gansu Agricultural University, Lanzhou, 730070 China; 2 College of Animal Science and Technology, Northwest A&F University, Yangling, 712199 China; 3 Gansu Academy of Agricultural Sciences, Lanzhou, 730070 China; 4 Ralco Nutrition Inc., Marshall, MN 56258 USA; 5 Furst-McNess Company, Freeport, IL 61032 USA

## Abstract

Essential oils (EO) are secondary metabolites usually made up of terpenoids and phenylpropanoids and have antimicrobial properties. However, the feeding effects of EO-Cobalt (EOC) on the performance of goats are largely unknown. Herein we investigated and reported the effects of dietary EOC (0, 52, and 91 mg daily) on fiber producing cashmere goats. We determined the resulting phenotypes including live growth, carcass weight, meat quality, and cashmere fiber traits. We show that dietary supplement of EOC significantly promoted average daily gain (*P* < 0.05), and significantly improved carcass weight, and meat and hair fiber quality (*P* < 0.05). We further conducted RNA-seq using skin and liver tissues from each group to assess the molecular mechanism conferring these phenotypic changes. A total of 191 differentially expressed genes were found in the skin tissues (0 vs 91 mg), while 1,127 DEGs were found in livers. Analyses of liver samples for differential gene action and functional prediction found that EOC stimulated physiological changes in the body’s immune system at both blood and cell levels. Our results demonstrated the potential of using EO-based feed ingredient to improve animal growth performance, meat quality and fiber quality, and further illustrated the molecular basis that contribute to phenotypes at physiological levels.

## Introduction

Essential oils (EO) are volatile aromatic compounds produced by plants (herbs and spices) as complex mixtures of secondary metabolites, EO have a large number of beneficial properties on health through their well-known antioxidant and free radical scavenging activities^1^. An active component in essential oils (EO) has been reported to possess high antifungal, antioxidant and antimicrobial activity^2^. Antimicrobial activity contributes to promoting a dynamic balance microflora population in the rumen and the lower gut which contributes directly to gut barrier integrity, reduced inflammation, improved competitive exclusion of pathogenic bacteria resulting in a stronger immune system. Essential oils appeared to be very promising compounds as they selectively reduced methane production and protein breakdown in both *in vitro* and *in vivo* studies^3^, while the use of EO as feed additives was accompanied with decreased feed degradability and lowered volatile fatty acid^4^. Previous studies have found that dietary incorporation of oregano essential oil (OEO) exerted strong antioxidant effects retarding lipid oxidation in meat during refrigerated and long-frozen storage so frozen stored meats have extended shelf life^5^. In addition, the study of dietary effects of OEO on pig performance, oxidative status and pork quality traits revealed that the meat, received higher organoleptic scores for color, taste, juiciness, and overall acceptance in both the blind and the labelled consumer tests^6^.

The antimicrobial action of essential oils in feed systems is well documented and there is a growing interest in studies of natural additives as potential antioxidants and as substitutes for antibiotics when illegality for antibiotic use is totally enforced worldwide^7^. Many sources of antioxidants of plant origin have been studied in recent years. Among these, the antioxidant properties of many aromatic plants and spices have shown to be effective in retarding the process of lipid peroxidation in oils and fatty foods^8,9^. Previous studies have found that EOC is beneficial in alleviating transportation stress and improving antioxidant activity^10,11^. In addition, Cobalt is the activator of various enzymes in the body, mainly involved in the synthesis of vitamin B_12_ in the form of Co^3+^. A small amount of cobalt can enhance the reproductive capacity of ruminants, Lack of cobalt often lead to anemia and dysplasia, livestock will appear low birth rate, reduced milk, young chicks born weak, low rate of weaning survival^12,13^.

Mammalian hair is produced by hair follicle (HF) cell proliferation and differentiation, the formation of HF is associated with epidermal and mesenchymal cells^14,15^. HF are unique to mammals, with a high degree of self-renewal ability, the only lifelong growth of organs. The studies of HF related to the mechanism at the transcriptional level of shows that many factors and their receptors plays an extremely important role in the regulation HF as an important link in the regulation of genes and environment *in vivo*^16^. RNA-seq was sequenced using high-throughput sequencing techniques for reverse transcription of all RNA’s in tissues or cells^17^, and useful for studying animal production and health and have become important components of systems genomic or systems biology methods^18^. The objective of the present study was to identify potential regulatory genes in supplementation with EOC for cashmere goats by characterizing the skin and liver transcriptome based on RNA-seq technologies. And this study reports important finding regarding potential regulatory genes and effects of EOC, including hair fiber and meat quality in farm animals.

## Material and Methods

### Ethical approval

All animal handing protocols in this study were approved by the Gansu Agricultural University Animal Care and Use Committee guidelines (approved ID: 2012-2-159), in compliance with the Regulations for the Administration of Affairs Concerning Experimental Animals (The State Science and Technology Commission of P. R. China, 1988).

### Experimental design and animal management

**Figure 1:**
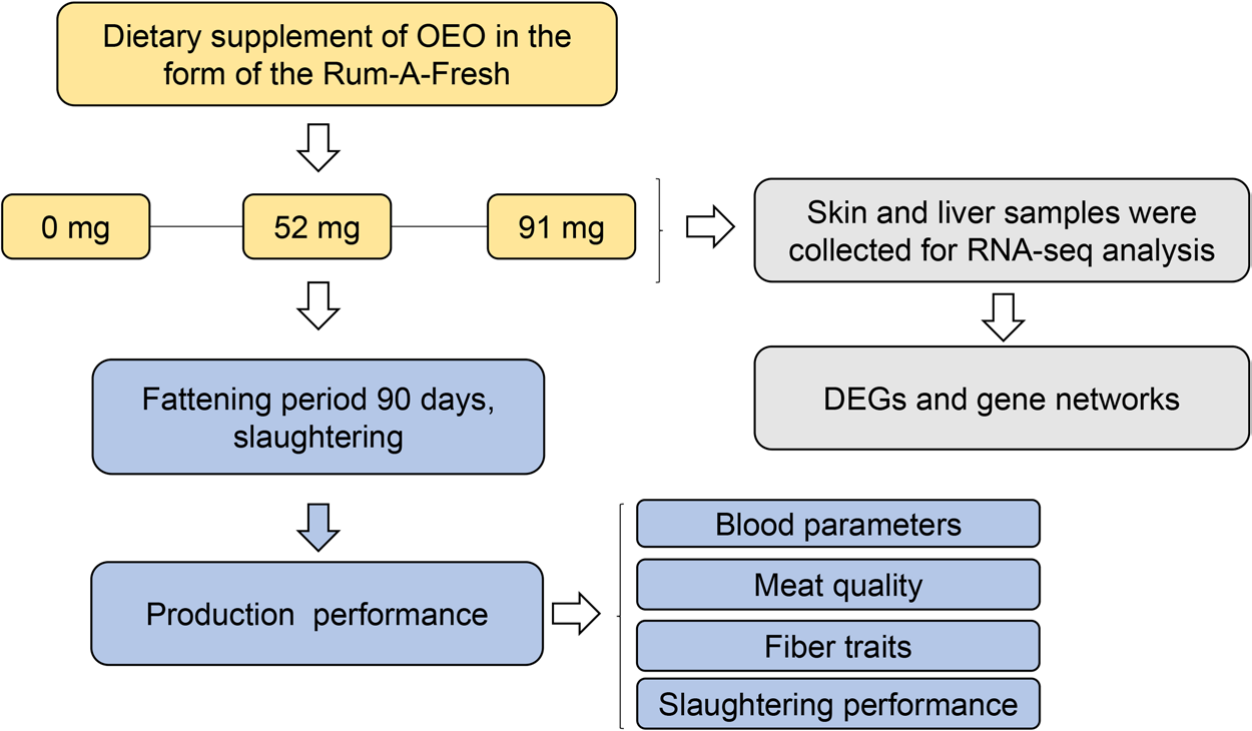
Study design of the present study.

A total of 45 castrated male cashmere goats were randomly divided into three groups. The cashmere goats in each group were animals of similar live weight (average weight 33.73 ± 2.11 kg) at fasting. There was no significant difference in body weight between three groups (*P* > 0.05). the EO and Co are added as product of Rum-A-fresh (RAF), the organic cobalt level is 0.75% in the RAF product. the essential oil amount is 1.3% of the RAF product. For each group, 0, 52, and/or 91 mg per head/day of EOC were added in daily diet in the form of an essential oil, lactic acid, and cobalt carbonate with clinoptilolite used as an ingredient dispersant. The fattening period lasted 90 days and was divided into three stages. Each fattening period was 30 days. Each test group of goats was evaluated for 90 days. There was a 15-day pre-feeding adjustment period at the beginning of fattening period. Uniform live body weights were obtained following a 12-hour fast. This was done to eliminate variation in body weight that might be related to weight changes associated with potential drinking or eating immediately before weighing. During the trial period, the goats were allowed to freely drink water, and feed twice every day. Table S9 shows the chemical composition of the commercial diet for each group. Three goats from each group were randomly selected for slaughtering at day 90. All goats were slaughtered in an accredited abattoir using carbon dioxide to stun the animal. The specific experimental design is shown in Fig. 1.

## Analytical Methods

### Analysis of growth and slaughter performance

The weight of goats was determined before morning feeding at day 0 and day 90, to calculate the average daily gain (ADG). Animals were then processed following the standard industrial techniques. For each animal, the weight of pelt, full gastrointestinal liver, spleen, left kidney tract, heart, and lungs were collected. Hot carcass weight was then recorded in order to evaluate hot dressing percentage as a ratio of hanging carcass weight and live weight.

### Analysis of meat quality

The meat quality was determined from the sample removed from the *Longissimus dorsi* (LD) muscle after carcass dissection, with no external fat or connective tissue. The measurement of pH, lightness (L*), redness (a*), yellowness (b*) were measured. Water loss rate, drip loss, cooking losses, and tenderness were conducted according to a previous study^19^. The first pH reading (pH1) was taken *in situ* on the LD muscle of the right side between the 4^th^ and the 5^th^ lumbar vertebra using penetrating electrode and a portable pH meter. Drip losses and other cooking losses were performed on *LD* muscle as described by Honikel^20^. The detailed method steps refer to a previous report^19^. The *LD* muscles were extracted according to the method of Folch^21^.The one-step extractive methylation procedure for fatty acids gain was performed in a gas chromatograph (Trace DSQGC/MS) with a capillary column (HP-5MS) using margaric acid (C17:0) as an internal standard. The oven temperature was programmed to provide three consecutive ramps, the first had an initial temperature of 120 °C maintained for 1 min then increased by 10 °C/min until it reached 175 °C, where it was maintained for 10 min; the second increased by 5 °C/min until it reached 210 °C, where it was maintained for 5 min and the third ramp increased by 5 °C/min to 230 °C, where it was maintained for 5 min. The carrier gas was helium at a flow rate of 2 mL/min. An automatic split less injector with a 1/50 split and a temperature of 250 °C was used. The injection volume was 1 μL. A flame ionization detector (FID) was used with an air flow of 450 mL/min, hydrogen flow of 40 mL/min and a detector temperature of 280 °C. Fatty acids were expressed in gravimetric concentrations (mg/g of freeze dried sample)^22,23^.

### Analysis of serum physiological and biochemical indexes

Jugular vein blood was collected from the goats 1 hour before slaughtering, the blood was preserved in the vacuum tube containing coagulant, blood was kept at 4 °C for 3 h, and then centrifuged (4000 r/min for 10 min at 4 °C) to extract the serum. The serum was used to determine blood physiological parameters.

### Skin staining

Skin tissues were used for histological sectioning, the procedures of histological sectioning according to the method of Carter and Clarke^24^. skin samples were extracted from the different goats and placed in tubes containing 4% paraformaldehyde solution (made with 0.1 M sodium phosphate buffer, pH = 7.4). After the following steps are embedding, cutting into slices, baking slides, H&E straining, mounting^25^.

### Total RNA isolation, library construction and sequencing

Total RNA was isolated from skin and liver samples using Trizol Reagent (Invitrogen) according to the manufacturer’s instructions. The quality and concentration of the total RNA were determined using an Agilent 2100 Bioanalyzer (Agilent). RNA samples were stored at -80 °C for later library construction and sequencing. Nine RNA libraries for each skin sample were constructed. Oligo (dTs) were used to isolate poly (A) mRNA. The mRNA was fragmented, and reverse transcribed using random primers. Second-strand cDNAs were synthesized using RNase H and DNA polymerase I. The double-strand cDNAs were then purified using the QiaQuick PCR extraction kit. The required fragments were purified via agarose gel electrophoresis and were enriched through PCR amplification. Finally, the amplified fragments were sequenced using Illumina HiSeq™ 2000 (Novogene, Beijing, China) according to the manufacturer’s specifications.

### Mapping reads to the reference genome

The original sequencing-received image data were transferred into sequence data via base calling, which is defined as raw data or raw reads stored in the appropriate format. Raw reads of all nine samples were pre-processed through the removal of containing adaptors-read with more than 17% unknown nucleotides (the minimum read length is 400 bp). The clean reads of each stage were aligned to the goat genome assembly (ARS1). At the same time, STAR add parameters of out Filter Intron Motifs Remove Non-canonical, In-the- post-sequences, so the resulting bam file supports the use of Cuffdiff^26^.

### Expression annotation

For gene expression analysis, the number of unique-match reads was calculated and normalized to FPKM (Fragment Per Kilo base of exon model per Million mapped reads). Expression levels of each gene between two groups were compared to give an expression difference using Cuffdiff as outlined by Trapnell^27^. The *P* value corresponded to differential gene expression at statistically significant levels (*P* < 0.05)^28^. False Discovery Rate (FDR) was used to determine the *P* value threshold. DEGs were defined as FDR ≤ 0.05 and absolute value of |log (fold change)| >1. Functional classification of the DEGs was performed using WEGO software. The KEGG (Kyoto encyclopedias of genes and genomes) pathway annotation was carried out using DAVID 6.7^29^.

### Statistical analysis

The analyses associated with RNA-seq were analyzed using R. We performed t-test using a one-way ANOVA with Dunnett’s post-hoc comparison procedure of the statistical analyses software SPSS version 22.0 to separate statistical means and to establish the probabilities of larger ‘P’ values.

## Results

### Finishing performance and carcass characteristics

The growth performance results from the goats during the finishing phases are shown in Table 1. The initial body weights by treatment were not significantly different (*P* > 0.05). The final body weight and ADG was affected by the feed levels; consequently, means of two treatments of 0 mg group and 91 mg group were significant different at the 5% level of probability (*P* < 0.05). The feed conversion rate (FCR) were significant increase in EOC addition group (*P* < 0.05). The meat marbling score of the 91 mg group was significantly higher also at the 5% level of probability than that of the control group (*P* < 0.05). Carcass weight, dressing percentage, net meat percentage, meat-bone ratio, and perineal fat are significantly different when contrasting the 91 mg group mean with the control group mean (*P* < 0.05).

**Table 1:** Effects of the EOC on growth and slaughter performance of goats.

Item	Group			SEM	*P*-value
	0 mg	52 mg	91 mg		
Initial body weight (kg)	33.38	33.41	34.38	0.49	0.95
Final body weight (kg)	42.05^a^	48.91^b^	49.66^b^	1.23	0.50
Average daily gain	0.09^a^	0.17^b^	0.17^b^	0.01	0.51
Feed conversion efficiency (%)	11.14^a^	31.69^b^	19.77^b^	3.40	0.03
Marbling score	2.53^a^	4.33^b^	4.00^b^	0.19	0.00
Longissimus Doris (g)	556.3	584.57	625.01	15.38	0.19
Carcass weight (kg)	20.23^a^	24.19^b^	23.9^b^	0.59	0.00
Dressing percentage (%)	49.00^a^	51.27^ab^	51.56^b^	0.50	0.07
Net meat percentage (%)	33.57^a^	34.07^ab^	35.98^b^	0.45	0.07
Meat-bone radio (%)	34.55^a^	30.38^a^	28.66^b^	0.89	0.01
Perirenal fat (kg)	0.92^a^	1.40^b^	1.52^b^	0.09	0.01
Left half carcass muscle area (cm^2^)	19.38	19.88	22.75	0.87	0.25

SEM = standard error measurement. ^a,b^Means with different superscripts differ significantly (*P* < 0.05).

### Meat quality

The muscle quality traits are shown in Table 2. The pH1 and pH24 were not affected by the feed levels, the cooked meat percentage was significantly affected by the EOC (*P* < 0.05), the cooking loss. water-holding capacity and drip loss are not significantly different when comparisons of the 91 mg group and the control group are considered (*P* > 0.05). The fatty acid composition of LD muscle is shown in Table 3, Saturated hypercholesterolemia FA (14:0) was significantly affected by EOC (*P* < 0.05). The contents of polyunsaturated fatty acid (PUFA), monounsaturated fatty acid (MUFA), saturated fatty acid (UFA) and saturated fatty acid (SFA) were not significantly influenced by the EOC (*P* > 0.05).

**Table 2:** Effects of the EOC on quality traits of goat’s LM.

Item	Group			SEM	*P*-value
	0 mg	52 mg	91 mg		
pH1	6.62	6.61	6.54	0.07	0.90
pH24	6.25	6.23	6.36	0.11	0.91
L*	49.94	49.71	50.16	0.18	0.67
a*	8.46	4.71	5.51	0.81	0.13
b*	12.99	12.51	12.82	0.16	0.57
Cooking loss (%)	14.69	15.84	18.09	1.36	0.65
Water-holding capacity (%)	5.56	8.24	4.79	1.08	0.45
Cooked meat percentage (%)	25.71^b^	31.38^b^	40.72^a^	2.53	0.02
Drip loss (%)	5.52	4.55	6.93	0.75	0.50

SEM = standard error measurement. ^a,b^Means with different superscripts differ significantly (*P* < 0.05).

**Table 3:** Effects of the EOC on total fatty acid concentration (mg/g muscle dry matter) and composition (g/100 g total FA) composition of goat’s LM.

Item	Group			SEM	*P*-value
	0 mg	52 mg	91 mg		
C14:0	1.34^a^	1.61^a^	0.32^b^	0.05	0.04
C16:0	20.02	21.94	21.21	0.36	0.09
C18:1 9t	2.15	1.79	1.67	0.11	0.19
C18:1 9c	51.01	50.74	51.78	0.49	0.70
C18:1 10c	1.99	2.08	2.04	0.05	0.84
C18:1 11c	0.27	0.34	0.25	0.03	0.47
C18:1 12c	0.14	0.19	0.14	0.01	0.45
C22:1n9	0.47	0.32	0.55	0.04	0.17
Total acid in muscle (mg/g)	121.70^b^	216.91^a^	107.67^b^	20.23	0.04
Polyunsaturated fatty acid (PUFA)	3.09	2.93	3.44	0.16	0.44
Monounsaturated fatty acid (MUFA)	58.29	57.62	58.52	0.52	0.78
Unsaturated fatty acid (UFA)	61.38	60.56	61.96	0.61	0.67
Saturated fatty acid (SFA)	38.61	39.44	38.03	0.61	0.67

SEM = standard error measurement. ^a,b^Means with different superscripts differ significantly (*P* < 0.05).

### Serum physiological and biochemical analyses

The contents of calcium, magnesium, creatinine, carbon dioxide-combining power, bilirubin acid, total protein and albumin in the 91 mg group were significantly higher than the control group at the 5% level of probability (*P* < 0.05) (Table 4). The contents of lactate dehydrogenase decreased with the addition of EOC, and the other means are not significantly different as indicated by a probability level greater than 5%.

**Table 4:** Effects of the EOC on serum physiological and biochemical of goat.

Item	Group			SEM	*P*-value
	0 mg	52 mg	91 mg		
Leukocyte (10^9^/L)	16.06	16.5	15.85	0.6	0.91
Erythrocyte (10^12^/L)	4.73	4.93	4.91	0.15	0.85
Hemoglobin (g/L)	144.16	138.83	147.5	2.18	0.27
Lymphocyte percentage (%)	55.41	53.51	54.1	1.6	0.89
Calcium (mmoL/L)	2.35^a^	2.43^b^	2.46^b^	0.01	0.01
Magnesium (mmol/L)	1.04^a^	0.93^ab^	0.88^b^	0.03	0.06
Phosphorus (mmol/L)	2.48	2.36	2.01	0.1	0.10
Ferrum (mmol/L)	38.41	28.81	32.21	2.3	0.23
Creatinine (umol/L)	63.13^a^	46.23^b^	62.13^b^	2.79	0.01
Carbon dioxide-combining Power (mmol/L)	20.88^a^	25.35^b^	24.96^b^	0.74	0.01
Bilirubin acid (umol/L)	3.23^a^	1.88^a^	2.33^b^	0.2	0.01
Total protein (g/L)	76.48^a^	71.06^ab^	73.13^ab^	0.88	0.03
Albumin (g/L)	35.50^a^	32.95^a^	33.73^b^	0.36	0.01
Globulin (g/L)	40.98	38.11	39.4	0.82	0.38
AST (u/L)	17.83	18	16	1.11	0.74
ALT (u/L)	89.5	73.16	85.66	3.75	0.18
γ-glutamyl trans peptidase (µ/L)	54.33	52	61.5	2.35	0.24
HDL (mmol/L)	2.02	1.8	2.16	0.09	0.25
Creatine kinase (µ/L)	197.16	235.5	233.83	12.08	0.36
Lactate dehydrogenase (µ/L)	372.66	367.66	342.33	19.94	0.82

SEM = standard error measurement. ^a,b^Means with different superscripts differ significantly (*P* < 0.05).

### Characters of Hair fibers

**Figure 2:**
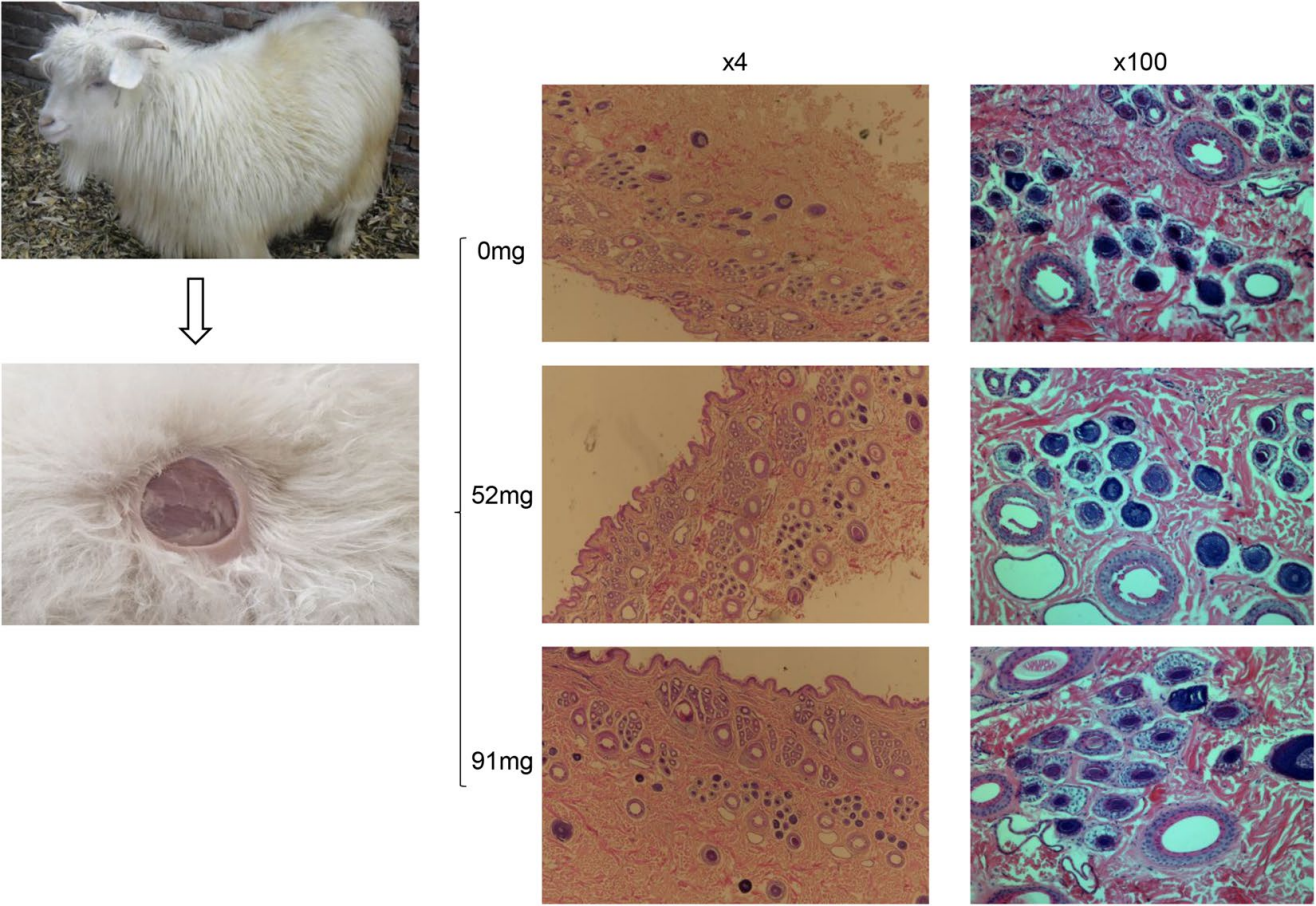
Longitudinal sections (×4 and ×100) H&E staining of cashmere goat skins.

The crack extension, EYS1.5 and elongation per unit length of the different treatment groups were significantly different (*P* < 0.05) (Table 5). Hair fineness was considered a very important economic trait of the cashmere goat included in the study reported herein. With the addition of essential oils, fineness tended to be thinner, but differences were not significant between the treatment groups (*P* > 0.05). In addition, the primary and secondary HF diameter was significant different (*P* < 0.05). Also, the diameter ratio of primary HF number to secondary HF (S/P ratio) was significantly different (*P* < 0.05) (Fig. 2).

**Table 5:** Effects of the EOC on hair fibers characters of cashmere goat.

Item	Group			SEM	*P*-value
	0 mg	52 mg	91 mg		
Degree of curling	1.39	1.44	1.6	0.05	0.31
Recovery rate	78.13	75.92	75.44	2.04	0.86
Crack extension (mm)	3.94^a^	5.21^b^	5.02^b^	0.21	0.02
Intension (CN/dT)	25.41	23.97	19.1	1.92	0.40
EYS1.5 (mm)	3.05^a^	4.29^b^	4.22^b^	0.23	0.04
Elongation per unit length (%)	39.41^a^	52.11^b^	49.82^b^	2.17	0.02
Fineness degree	17.99	20.19	20.08	0.52	0.16
Natural length (cm)	3.53	4.11	3.64	0.30	0.72
Level of stretch (cm)	4.04	5.02	4.38	0.29	0.42
Whiteness	26.04	33.11	41.01	4.02	0.33
Primary HF diameter (mm)	314^a^	335^a^	365^b^	7.43	0.01
Secondary HF diameter (mm)	94^a^	117^b^	120^b^	4.60	0.03
S/P	9.37^a^	10.8^b^	14.65^b^	1.06	0.04

SEM = standard error measurement. ^a,b^Means with different superscripts differ significantly (*P* < 0.05).

### Transcriptome profiling of 0 mg group, 52 mg group and 91 mg group of skin and liver

With regard to dietary supplementation of EOC affects, the growth performance, physiological, and biochemical indexes of the blood and the meat quality of the cashmere goats were affected. To elucidate the consequences these changes at genetic level, skin and liver were analyzed for RNA-seq.

To quantify the gene expression patterns of skin and liver tissues from these three groups, we constructed three RNA-seq libraries and then subjected them to deep sequencing using Illumina HiSeq. 2000. In total, in liver samples, we obtained 52,343,437, 58,562,606 and 68,157,431 reads from 0 mg,52 mg and 91 mg group, respectively, among them, 50,670,031, 56,320,741, and 65,948,002 short reads could be mapped to the goat reference genome (Table S1). In skin samples, researchers in this study obtained 63,703,887, 66,245,924 and 55,055,371 reads from 0 mg, 52 mg and 91 mg group, respectively. Among them, 61,632,490, 64,065,321, and 53,495,041 short reads could be mapped to the goat reference genome. Of the total reads, the rate of match reads was more than 96%, and the remaining reads were unmatched (Table S2). Of the total reads, the rate of match reads was more than 96%, and the remaining reads were unmatched. Expression levels of a distinct gene from two groups were compared to give an expression difference using the Cuffdiff (Table S1). The number of DEG in skin_0 mg, skin_91 mg. liver_0 mg, liver_91 mg, respectively, for transcripts detected with |log (fold change)| >1 and q value < 0.05, A total, 1127 DEGs were found in liver_0mg vs liver_91mg, 191 DEGs were found in skin_0 mg vs skin_91 mg.

### DEGs in goat skin and liver (0 vs 91 mg)

**Figure 3:**
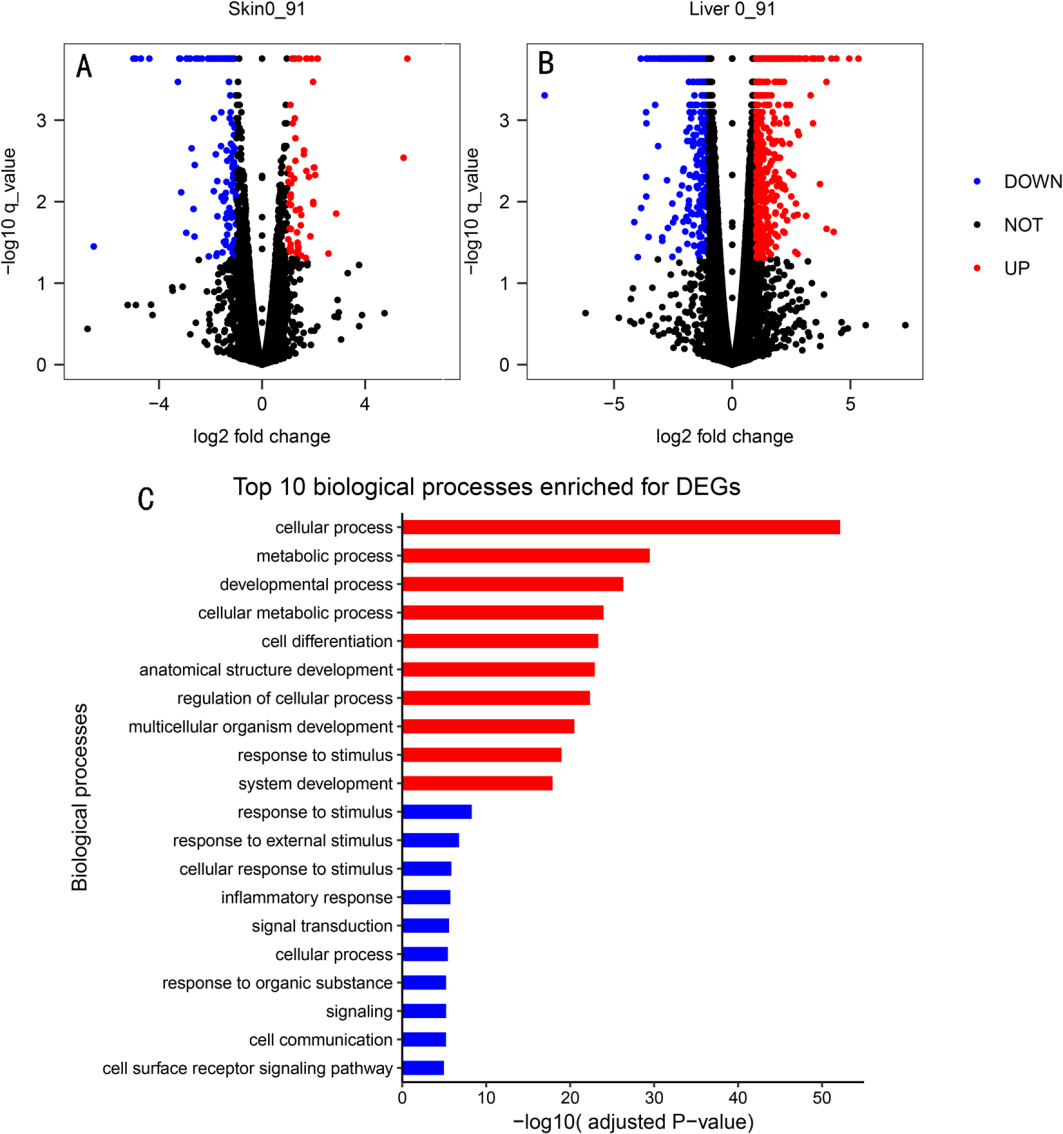
(**A**) Schematic representation of the differentially expressed genes between 0 mg group and 91 mg group of skins. (**B**) Schematic representation of the differentially expressed genes between 0 mg group and 91 mg group of livers. (**C**) Top 10 biological processes enriched for DEGs. Red bars indicate biological process enriched by DEGs in liver, blue bars indicate biological process enriched by DEGs in skin.

In skin samples, we identified 190 genes that were expressed at least two-fold differently between the two types of EOC (Table S3). 58 genes from 91 mg were upregulated compared with 0 mg and 132 were downregulated (Fig. 3A). In liver samples, we identified 1125 genes that were expressed at least two-fold differently between the two types of EOC. 613 genes from 91 mg were upregulated compared with 0 mg and 512 were downregulated (Fig. 3B). The specific genetic information is shown in Table S4.

### GO-term analysis of DEGs

**Figure 4:**
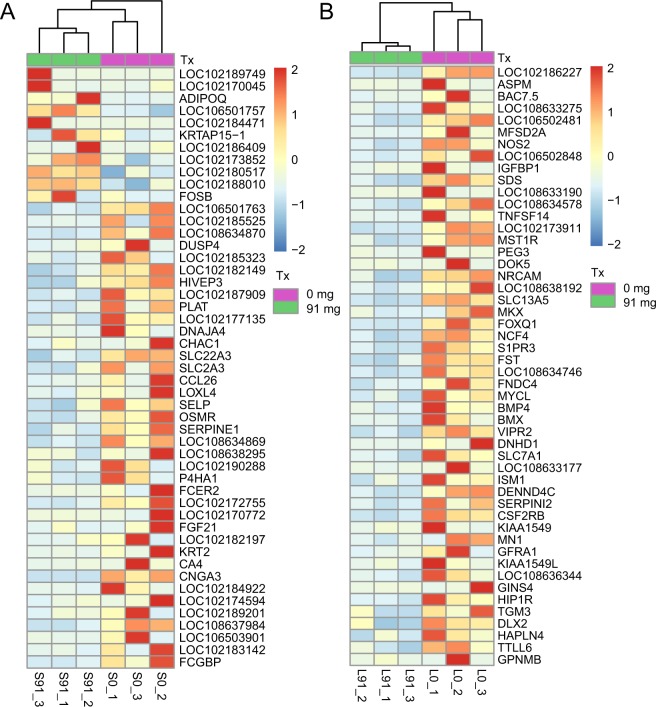
The FPKM heatmap of differentially expressed genes between 0 mg group and 91 mg group of skin (**A**) and liver (**B**).

In skin samples, 192 DEGs were categorized into three gene ontology categories: cellular component, biological process and molecular function. The top five functional categories of DEGs in 0 mg vs 91 mg included cell, cell part, binding, cellular process, biological adhesions (Fig. S1). The top five biological processes enriched for DEGs included response to stimulus, response to external stimulus, cellular response to stimulus, inflammatory response, signal transduction. (Fig. 3C). Under the cellular component category, a large number of up-regulation DEGs, as well as down-regulation DEGs, was categorized as extracellular region, for example, the *FGG*, *MUCL1*, *ADIPOQ*, *ASGR1*, *AGT*, *TNFRSF11B*, *TNFAIP6*, *COL6A5*, *HSPA6* and *EGFL6* were enriched in the extracellular region and *SYNDIG1* was enriched in the synapse (Fig. 4).

In liver samples, 1,127 DEGs were categorized into three gene ontology categories: cellular component, biological process and molecular function. The top five functional categories of DEGs in 0 mg vs 91 mg included cell, cell part, binding, cellular component organization, biological regulation (Fig. S1). The top five biological processes enriched for DEGs included cellular process, metabolic process, developmental process, cellular metabolic process, cell differentiation (Fig. 3C). Under the cellular component category, a large number of up-regulation DEGs, as well as down-regulation DEGs, was categorized as extracellular region, for example, the *TSKU*, *LYPD8*, *SEMA3C*, *PLA2G2F*, *MMRN1*, *TFF2* and *IHH* were enriched in the extracellular region (Fig. 4).

### KEGG pathway analysis of DEGs

In order to explore the mechanism of EOC, we performed KEGG pathway analysis of the dysregulated genes between 0 mg and 91 mg. In liver samples, the results indicated that “MAPK signaling pathway” (26 DEGs, 8.07%) was the first highly significantly enriched (*P* value = 0.001), the MAPK signaling pathway promotes cell survival by a dual mechanism comprising the posttranslational modification and inactivation of a component of the cell death machinery and the increased transcription of pro-survival genes^30^; “Rap1 signaling pathway” (18 DEGS, 5.59%) was the second significantly highly enriched (*P* value = 0.03), the Rap1 is activated by extracellular signals through several regulatory proteins, and it might function in diverse processes, ranging from modulation of growth and differentiation to secretion, integrin-mediated cell adhesion and morphogenesis (Fig. S2)^31^.

## Discussion

### Improvement of productive traits with dietary supplementary of EOC

In this study, we show that diets supplemented with EOC are capable of significantly improve production performance including animal growth, carcass weight, physiological parameters, meat quality, and fiber traits in cashmere goats.

The addition of EOC significantly increased FCR, resulting in increased ADG of goats (Table 1). The main reason is that EOC was used as a feed additive derived from plants. It has a spicy, aromatic taste, stimulates appetite, and increases feed intake^32^, and thereby resulted in increased ADG and improved finishing and carcass performance. Previous studies reported that EO is able to significantly improve FCR, for instance, an inclusion level of 400 mg/kg of EO enhanced FCR by approximately 12%^33^. In line with previous effects to improve meat traits with essential oils, it is plausible that addition of EOC enhanced ADG and eventually promoted finishing and carcass performance in goats.

In terms of blood physiological and biochemical indexes, the change of nutritional level of diets may cause metabolite changes related to available nutrients circulation in blood. For example, the addition of EOC significantly increased the proportion of calcium in the blood, reducing the proportion of magnesium (Table 4), calcium and phosphorus are essential mineral elements in animals and play important roles in maintaining the body’s acid-base balance and regulating osmotic pressure^34^. These changes can also affect the efficiency of growth and development of food and fiber producing animals and their subsequent products in food and fiber output. The effects of diets on the growth and development of goats were studied by measuring the changes of serum biochemical indexes considering dietary nutritional levels, which was of great significance to the preparation of diets in goat breeding practices. Blood biochemical indicators reflect the healthy status of growth and development in goats. This is an important measurement of goat body conditions^35^. Serum protein concentration reflects the feed status and animal physiology and growth and development level. The results of this study showed that the total protein levels in the 91 mg and 52 mg groups were significantly higher than those in the 0 mg group (*P* < 0.05). This may be due to EOC promotion of increased liver synthesis as serum total protein levels increased.

We further found that the perirenal fat and marbling score significantly increased (*P* < 0.01), indicating that the addition of EOC significantly enhanced the fat deposition. Further examination of cooked meat percentage found that the addition of EOC significantly improved the quality traits of LM. Our results show that the addition of EOC to feeds can alleviate the tendency of muscle acidification caused by muscle glycolysis and reduce the possibility of PSE meat formation. After adding EOC, the muscle water loss was significantly reduced, and the water retention performance of muscles was significantly improved. This would be beneficial to maintaining tenderness, juiciness, quality of meat products, and an improved meat quality^36^. Previous study also suggested that the addition of 1000 ppm OEO to pig diet could be recommended to produce meat of good quality and minimum lipid oxidation^37^. Additionally, water holding capacity is an important meat quality indicator. It refers to the muscles by external forces such as pressure, heating, freezing, chopping to maintain water capacity. Water holding capacity directly affect the flesh, tenderness and nutritional value^38,39^. In the present study, 91 mg group and 52 mg group of cooked meat rate was significantly higher than 0 mg group (*P* < 0.05), with water loss and cooked meat rate to measure the mutton water holding capacity whereas the lower the water loss, indicated higher rates of meat and water retention suggesting improved meat quality related to water retention of mutton^22^. We showed that the effects of the EOC on total fatty acid concentration and composition, the results found that the myristic (C14:0) was significant increased with EO supplementation (Table 3). It is reported that medium-chain FA (*e*.*g*. myristic) biosynthesis relys on low activity and mRNA abundance of lipogenic enzymes (*e*.*g*. acetyl CoA carboxylase and fatty acid synthase)^40^, we assume that the decrease of C14:0 may results from the inhibition of acetyl CoA carboxylase and fatty acid synthase after dietary EOC supplementation. Moreover, the flavor in meat is related to stearic (C18:0) and linoleic (C18:2) acid levels^41^, we show that the addition of EOC has not influenced meat flavor in this study (Table 3). The FA composition of ruminant edible fats is mostly determined by complex interactions between dietary factors and rumen metabolism^42^. The effect of the EO supplementation on meat FA profile recorded in the current study is in agreement with the results of previous study^43^, which observed that the supplementation of artemisia EO affected lamb meat FA profile but rosemary essential oils did not have effect. The present study also confirms the importance of EO by-products diet in protecting and maintaining higher FA levels in animal tissues due to their content in polyphenol compounds^44,45^. Therefore, EOC supplementary provides an alternative way to modify the FA composition of meat in food animals.

Early studies described that EOs, from slow-growing trees such as *C*. *obtuse*, have antimicrobial and antifungal effects^46^. Such EO was used in commercial hair care products since it promotes hair growth since a positive regulatory gene *VEGF* was involved^47^. EOs are found to enhance hair growth and promote HF proliferation^48,49^. In this study, the 91 mg group and 52 mg group exhibited an increased diameter of HFs and S/P ratios (Fig. 2 and Table 5), indicating that the addition of EOC also improved hair fiber output (*P* < 0.05). We assume that dietary supplement of EOC has a positive effect to dietary energy intake with increased ADG, and therefore resulted in cumulative hair diameter and HF density.

Taken together, we demonstrate that that the addition of EOC had a significant effect on the performance, meat quality, blood physiological and biochemical indexes and HF of tested goats. We designed the transcription analysis of skin and liver, to further study the important economic traits affected by the regulation of genetic networks of supplementation with EOC.

### Differentially expressed genes of liver samples

Liver plays an important role in regulating the supply of animal nutrients^50^. The liver transcriptomic profiles thus may lead to the identification of genes that are important for regulating feed efficiency^51^. In the 91 mg group, the top five upregulated DEGs were *NKG2D*, *HRASLS2*, *LOC102179132*, *ACTA1*, and *SIM1* (Table S7). Ectopic expression of *NKG2D* ligands causes rejection of transfected tumor cells by natural killer cells and primed cytotoxic T cells in syngeneic mice. Immunity is induced against subsequent challenges with tumor cells that lack NKG2D ligands^52^. The NKG2D-ligand system, stimulation through the receptor can lead to the enhancement of innate immune functions, mediated by NK cells and myeloid cells, and the enhancement of adaptive immunity^53^. The *HRASLS2* gene belongs to the H-REV107 gene family involved in the regulation of cell growth and differentiation. *HRASLS2* is expressed at high levels in normal tissues of the small intestine, kidney, and trachea^54^. Arachidonates have esterified cholesterol, to increase blood vessel elasticity, reduce blood viscosity, and regulate blood cell function in a series of physiological activities^55,56^. Combined with GO-term analysis of DEGs, we found that these genes are mainly involved in the cellular process, metabolic process, developmental process, and cellular metabolic process, and cell differentiation metabolism (Fig. 3C). We hypothesize that the increase of feed conversion rates and improvement of meat quality, which may be related to the promotion of changes in these genes at the cellular and metabolic levels.

In the 91 mg group, the top five differential genes were significantly downregulated of TFF2 (Table S8), trefoil factor family (TFF) domain peptides consist of three members that play a role in intestinal mucosal defense and repair, and in tumorigenesis^57,58^. Recent study supported the notion that Tff3 peptide is included in the hepatic glucose and glycogen metabolism. Tff3 knockout animals had more glycogen positive cells and those cells had more glycogen accumulated in them when compared to wild type and Tff2 knockout animals^59^. We believe that the down-regulation of TFF gene may be related to energy metabolism in goats.

### Differentially expressed genes of skin samples

In the 91 mg group, the top three DEGs in skins were significantly upregulated of Odorant-binding, HRAS-like, BOLA. Odorant binding protein (OBP) is the major odorant binding component of mammalian nasal mucosa^60,61^. Previous investigators have verified that OBPs play a role in insect olfactory responses. Odor-binding proteins in the lymph fluid surrounding olfactory neurons play an important role in promoting the binding of odor molecules to odorant receptors and the sensitivity of olfactory responses. Combined with the GO-term analysis of DEGs, we found that these genes are mainly involved in the response to stimulus, response to external stimulus, cellular response to stimulus, inflammatory response, signal transduction (Fig. 3C). However, the physiological functions of OBPs are roughly summarized as follows: recognition and binding of specific odor molecules^62^; recognition and binding of specific odor molecules^63^; rapid inactivation of odor molecules that complete stimulation. This is consistent with GO analysis-related pathways.

HRAS-like is the same gene as the liver tissue upregulated gene. In the 91 mg group, the first two differential genes were significantly downregulated of lipopolysaccharide-binding, *MUCL1* (Table S5), lipopolysaccharide-binding was descripted lipopolysaccharide-binding, lipopolysaccharide binding protein may control the response to lipopolysaccharides under physiologic conditions by forming high-affinity complexes with lipopolysaccharides that bind to monocytes and macrophages, which then secrete tumor necrosis factor^64^. MUCL1 is an attractive tumor associated antigen as a potential therapeutic target. However, very little is known about the cellular location and biological functions of the MUCL1 protein^65^. Through these gene-related functions, we found that these genes play a role in the changes of the skin’s HF at cellular response to stimulus, inflammatory response, and signal transduction.

### Correlation analysis of MAPK and Rap1 signaling pathway

HF development and periodic growth process by a variety of signaling pathways. Some of these signaling molecules belong to the MAPK family. The genetic mutations, epigenetic modifications, and posttranslational modifications of ligands, receptors, intermediate signaling molecules, downstream transcription factors and their target genes in these signaling pathways affect the development of animal HF and lead to hair growth and hair quality changes^16^. The mitogen-activated protein kinase (MAPK) cascade is a highly-conserved module that is involved in various cellular functions, including cell proliferation, differentiation and migration^66^. We found that the addition of EOC led to an increase in the number of skin HF in cashmere goats, and the major regulatory genes were on the MAPK signaling pathway, and previous studies have shown that MAPK is an extracellular signal protein kinase transduction pathway, which Participate in pathogenesis of many skin diseases^67,68^.

In lower eukaryotes, Rap1 is implicated in processes that involve either the polarity of cells or the proper functioning of cells, rather than processes that are related to cell proliferation and differentiation. In mammalian cells, Rap1 has also been implicated in various cellular processes^31^. Rap1 is a small GTPase that controls diverse processes, such as cell adhesion, cell-cell junction formation and cell polarity^69^. Like all G proteins, Rap1 cycles between an inactive GDP-bound and an active GTP-bound conformation (Fig. S3). A variety of extracellular signals control this cycle through the regulation of several unique guanine nucleotide exchange factors (GEFs) and GTPase activating proteins (GAPs). Rap1 plays a dominant role in the control of cell-cell and cell-matrix interactions by regulating the function of integrin’s and other adhesion molecules in various cell types. Rap1 also regulates MAP kinase (MAPK) activity in a manner highly dependent on the context of cell types (Fig. S3). Therefore, the addition of EOC may activate RAP1 and MAPK signaling pathway at the level of cell differentiation, thus affecting the growth performance of goats.

## Conclusions

In summary, by investigating the effects of dietary EOC (0, 52 mg and 91 mg daily) in cashmere goats, we demonstrate the supplement of EOC resulted in a promoted average daily gain and improved phenotypes (cashmere fiber traits, carcass weight, and meat quality). RNA-seq using skin and liver tissues further revealed DEGs that are important to understand the molecular basis conferring the phenotypic changes. We found that EOC mainly stimulated the body’s immune system, including blood and cell levels related physiological changes.

## Electronic supplementary material

Dataset 3

Dataset 4

Supplementary File
