# Supplementary material for: Dietary supplementation with Essential-oils-cobalt for improving growth performance, meat quality and skin cell capacity of goats
Source: Sci Rep. 2018 Aug 2;8:11634. doi: 10.1038/s41598-018-29897-3 (PMC6072763; doi:10.1038/s41598-018-29897-3)
Supplement: Supplementary file 3 — Supplementary File [file 41598_2018_29897_MOESM3_ESM.pdf]

Dietary supplementation with Essential-oils-cobalt for improving growth performance, meat quality and skin cell capacity of goats

Zhaomin Lei[1], Ke Zhang[2], Chao Li[2], Jianping Wu[3], Delmer Davis[4], David Casper[5], Hui Jiang[1], Ting Jiao[2], Xiaolong Wang[2], Jianfu Wang[1]*

Supporting information

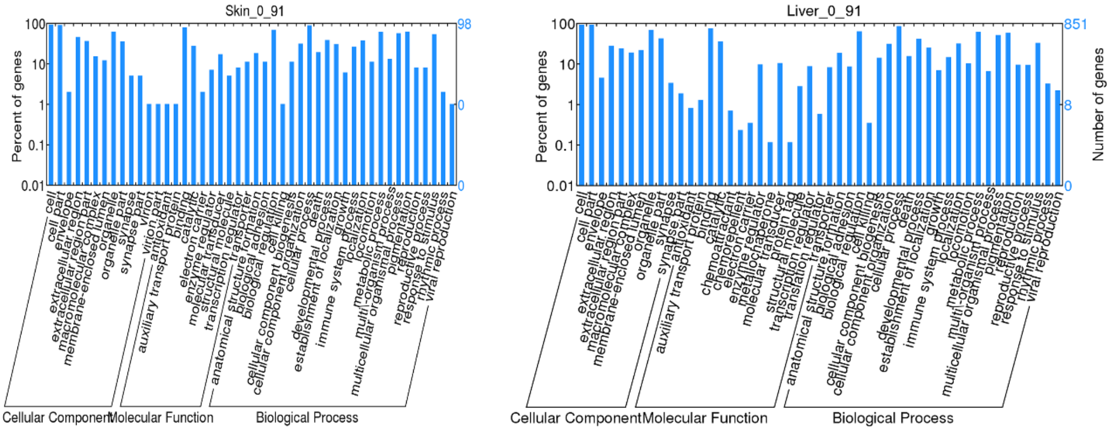

Figure. S1 Functional categorization of differentially expressed genes among libraries. The results are summarized in three main categories: biological process, cellular component and molecular function. The X-axis indicates the second level term of gene ontology; The Y-axis shows the percentage of genes.

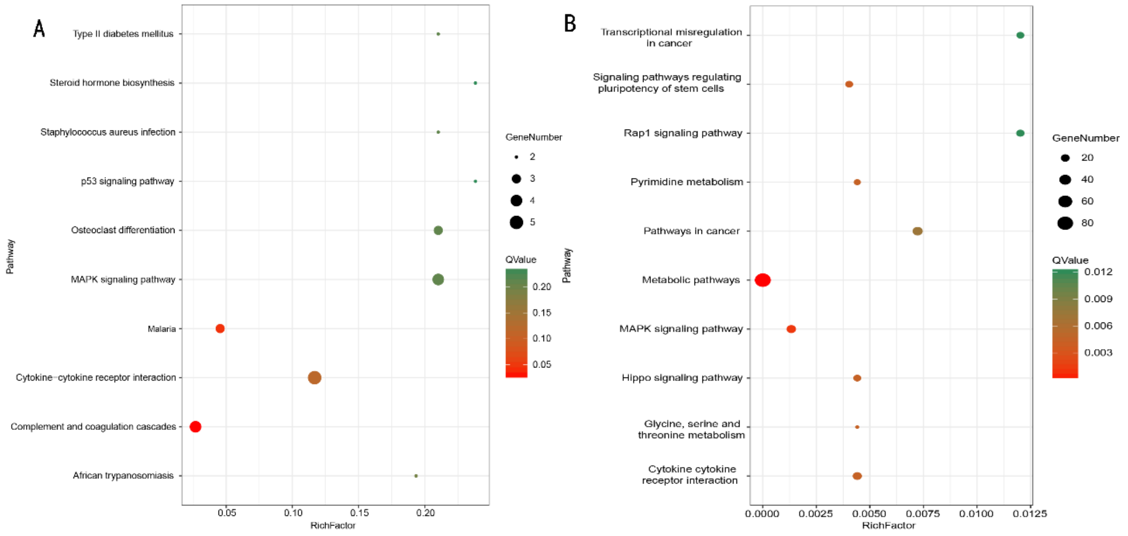

Figure. S2 Scatter plot for KEGG enrichment results of skin (A) and liver (B) samples. The top 10 enrichment pathways are shown in the senior bubble chart. The Rich factor is the ratio of DEGs numbers annotated in this pathway term to all gene numbers annotated in this pathway term. Q value is the corrected *P* value.

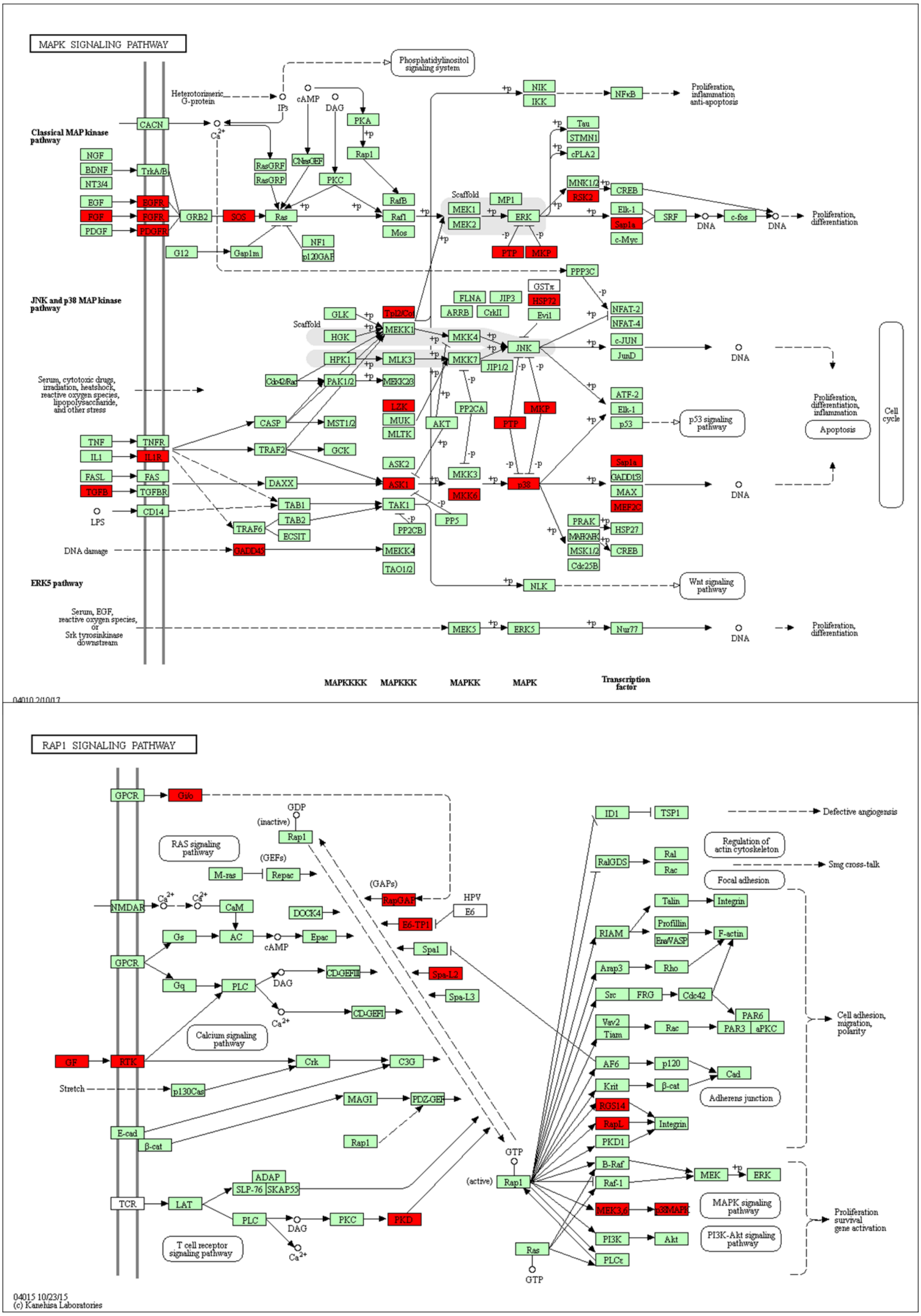

Figure. S3 DEGs related to fatty acid degradation and MAPK and Rap1 signaling pathway between 0g and 7g. KEGG pathway maps for (a) MAPK signaling pathway (has04010) and (b) Rap1

signaling pathway (hsa04015). Up-regulated genes are marked with red borders and down-regulated genes with green borders. Non-change genes are marked with black borders.

Table. S1 Summary of read numbers based on the RNA-seq data of liver sample from cashmere goat.

Item	L0_1mg	L0_2mg	L0_3mg	L52_1mg	L52_2mg	L52_3mg	L91_1mg	L91_2mg	L91_3mg
Total Reads	71344811	59799198	59967653	66797143	64142085	67798545	60794980	56235927	48135205
	69189949	57974454	57733067	64622495	62208665	65364803	58922140	54840505	46722479
Mapped Reads	96.98%	96.95%	96.27%	96.74%	96.99%	96.41%	96.92%	97.52%	97.07%
	2154613	1823876	2236793	2177587	1930677	2433968	1872485	1394651	1410362
Unmapped Reads	3.02%	3.05%	3.73%	3.26%	3.01%	3.59%	3.08%	2.48%	2.93%

Table. S2 Summary of read numbers based on the RNA-seq data of skin sample from cashmere goat.

Item	S0_1mg	S0_2mg	S0_3mg	S52_1mg	S52_2mg	S52_3mg	S91_1mg	S91_2mg	S91_3mg
Total Reads	50504358	45621130	60904822	46888448	68752121	60047249	69230283	66205765	69036246
Mapped Reads	49066428	44159884	58783780	45282446	65565079	58114697	66861393	64216283	66766330
	97.15%	96.80%	96.52%	96.57%	95.36%	96.78%	96.58%	97.00%	96.71%
Unmapped Reads	1439374	1459876	2119488	1608274	3190098	1933521	2367676	1986173	2271292
	2.85%	3.20%	3.48%	3.43%	4.64%	3.22%	3.42%	3.00%	3.29%

Table. S3 Gene with different expression in skin sample.

See in Excel Table S3

Table. S4 Gene with different expression in liver sample.

See in Excel Table S4

Table. S5 Downregulated genes in 91mg group of skin sample.

	Gene Expression (RPKM)		log2(fold_change)	
Gene	Skin-0	Skin-91	Skin-0/Skin-91	Description
LOC102186320	4.62629	0.0499379	-6.53358	lipopolysaccharide-binding
MUCL1	317.535	9.96511	-4.99389	mucin-like
LOC102186227	82.2486	2.74295	-4.90619	40S
AGT	4.52778	0.173776	-4.70351	angiotensinogen
COL6A5	7.59996	0.366286	-4.37495	collagen
LOC102172204	0.720661	0.0749077	-3.26614	ceruloplasmin
P2RX1	2.35987	0.254663	-3.21204	P2X
HSPA6	51.9299	5.72381	-3.18152	heat
ASGR1	1.23269	0.139917	-3.13917	asialoglycoprotein
IDO1	0.816633	0.106258	-2.94211	indoleamine
LOC102173911	118.266	15.4479	-2.93655	40S
NOX5	5.05322	0.672297	-2.91003	NADPH
LOC102179411	51.9794	7.46236	-2.80024	uncharacterized
LOC108634578	0.995586	0.144341	-2.78607	LOC108634578
LOC108638302	2.41848	0.36341	-2.73443	LOW
NCR3LG1	0.538649	0.0851759	-2.66083	natural
TNFRSF11B	0.543829	0.0883123	-2.62247	tumor
SLC35D3	0.464806	0.0759602	-2.61331	solute
TNFAIP6	17.6269	2.94539	-2.58125	tumor
LOC108634945	12.7195	2.28884	-2.47435	keratin-associated
FCGBP	53.2682	10.5671	-2.3337	IgGFc-binding
LOC102183142	4.17151	0.972418	-2.10092	T-cell
LOC102181854	1.17636	0.280574	-2.06788	platelet

LOC106503901	1.60708	0.388764	-2.04748	uncharacterized
LOC108637984	6.71869	1.67896	-2.00061	uncharacterized

Table. S6 Upregulated genes in 91mg group of skin sample.

Gene	Gene Expression (RPKM)		log2 (fold_change)	Description
	Skin-0	Skin-91	Skin-0/Skin-91	
LOC102189749	1.0311	51.3407	5.63785	odorant-binding
LOC108634205	0.0495323	2.23001	5.49254	HRAS-like
LOC102181347	0.0981548	0.720145	2.87516	BOLA
LOC102168566	0.126036	0.751021	2.57501	uncharacterized
LOC102170045	1.02439	4.58247	2.16136	BOLA
ADIPOQ	0.551636	2.43737	2.14354	adiponectin
LOC102168852	2.80413	11.6943	2.06019	LOW
LOC108637753	1.81102	7.39943	2.03061	uncharacterized
LOC102176897	0.406683	1.6363	2.00846	dihydrodiol
FGG	0.17205	0.681285	1.98543	fibrinogen
LOC108635541	0.697188	2.75951	1.98479	LOC108635541
MC4R	0.565168	2.22628	1.97788	melanocortin
LOC106501757	1.20879	4.57862	1.92135	NKG2D
MKRN2OS	0.465646	1.7064	1.87365	MKRN2
EGFL6	0.266072	0.936083	1.81482	epidermal
LOC102184471	14.9864	49.92	1.73597	uncharacterized
LOC102189387	0.743944	2.44715	1.71783	LOC102189387
KRTAP15-1	80.5302	263.844	1.71208	keratin-associated
LOC108637993	0.23083	0.749418	1.69894	phospholipase
LOC102171558	0.661759	2.04229	1.62581	BOLA
SYNDIG1	0.42388	1.30324	1.62037	synapse
SLC2A4	0.412609	1.18423	1.52111	solute
LOC108634131	0.648142	1.85656	1.51825	6-pyruvoyl

LOC102180551	0.740474	2.09553	1.50079	uncharacterized

Table. S7 Upregulated genes in 91mg group of liver sample.

Gene	Gene Expression (RPKM)		log 2 (fold_change)	Description
	Liver-0	Liver-91	Liver-0/Liver-91	
LOC106501757	0.703917	28.4919	5.339	NKG2D
LOC108634205	0.0844376	2.60532	4.94743	HRAS-like
LOC102179132	0.0529669	1.12097	4.40351	arachidonate
ACTA1	0.0736282	1.43823	4.28789	actin
SIM1	0.0503048	0.921255	4.19483	single-minded
RPRM	0.0922887	1.46339	3.98701	protein
LOC102174619	0.207069	3.27795	3.98461	cytochrome
IHH	0.900729	12.408	3.78403	indian
PYURF	2.61317	34.1682	3.70878	protein
FXYD2	5.68827	71.6753	3.65541	sodium/potassium-transporting
MMRN1	0.190982	2.15265	3.49461	multimerin-1
LOC102173971	3.40197	38.0387	3.48303	UDP-glucuronosyltransferase
LOC102187785	0.802529	8.60302	3.42222	cholesterol
SEMA3C	0.0436521	0.465449	3.4145	semaphorin-3C
RNF43	0.280991	2.90056	3.36773	E3
PLA2G2F	0.081797	0.813678	3.31434	group
LYPD8	0.336083	2.97703	3.14699	ly6/PLAUR
C25H16orf89	0.101644	0.887966	3.12698	UPF0764
TSKU	39.1141	338.28	3.11246	tsukushin
LOC102186942	0.954838	8.03271	3.07256	dihydrodiol
NECAB2	1.28146	10.546	3.04083	N-terminal

Table. S8 Downregulated genes in 91mg group of liver sample.

Gene	Gene Expression (RPKM)		log2(fold_change)	Description
	Liver-0	Liver-91	Liver-0/Liver-91	
TFF2	122.27	0.499679	-7.93485	trefoil
LOC102180785	3.45564	0.195683	-4.14236	proteoglycan
LOC102168305	1.61934	0.101457	-3.99647	LOC102168305
LOC102186227	58.2474	3.98272	-3.87037	40S
LOC102170003	7.17892	0.496758	-3.85315	cathelicidin-2
MPO	1.37248	0.10975	-3.64449	myeloperoxidase
BAC5	4.35669	0.348907	-3.64231	cathelicidin-2
CDHR4	0.873507	0.0702769	-3.6357	cadherin-related
LOC106502777	0.704532	0.0568662	-3.63102	uncharacterized
ASPM	0.498268	0.040265	-3.62933	abnormal
BAC7.5	5.77289	0.482711	-3.58006	Bac7.5
LOC108633275	13.5802	1.14315	-3.57043	tumor
LOC102180344	5.69432	0.488842	-3.54208	uncharacterized
LOC106502481	31.8637	2.90076	-3.45741	uncharacterized
MFSD2A	191.765	19.2054	-3.31975	sodium-dependent
NOS2	1.05043	0.107068	-3.29438	nitric
MAP28	8.13747	0.849211	-3.26039	MAP28
LOC106502848	16.2728	1.83083	-3.1519	uncharacterized
AZU1	2.82868	0.321727	-3.13622	azurocidin
IGFBP1	607.15	72.0544	-3.0749	insulin-like

Table. S9 Ingredients and nutrients of the experimental diets %.

Ingredients	Proportion %
Corn cob	12
Alfalfa	5
Rape straw	10
Wheat straw	10
Corn	48.6
Soybean meal	2
Rape seed meal	3
Cotton seed meal	6
Premix	1
Stone powder	0.9
NaCl	0.5
Baking soda	1
Total	100
Nutrient level	
Digestible energy (MJ/kg)	11.2
Crude protein (%) of DM	10.5
NDF (%)	38.4
Starch (%)	29.5
Ca (%)	0.58
P (%)	0.29

Note: The first stage of dry matter intake of 800 g; the second stage of dry matter intake 900 g; the third stage of dry matter intake of 1000 g. Formulated to provide (per kg of dry matter): S, 200 mg; Fe, 25 mg; Zn, 45 mg; Cu, 8 mg; Mn, 40 mg; I, 0.3 mg; Se, 0.2 mg; Co,0.1 mg; VA, 980 IU; VD, 120 IU; VE, 25 IU.
